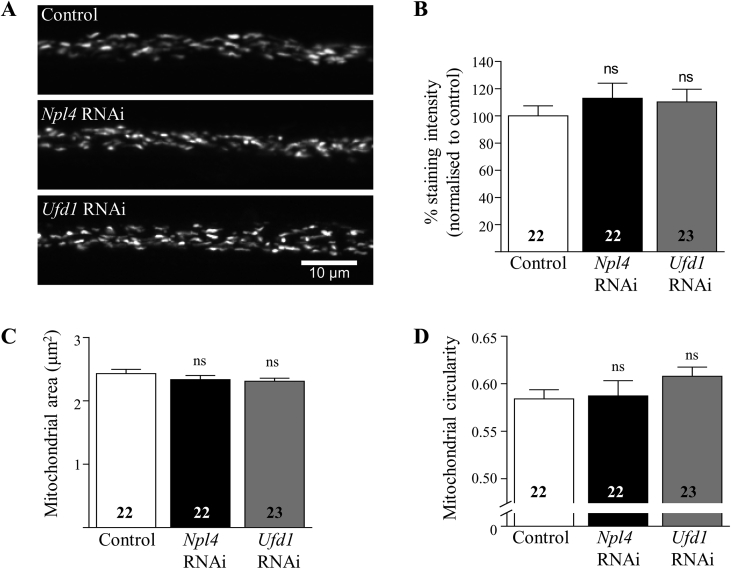# Roles for the VCP co-factors Npl4 and Ufd1 in neuronal function in *Drosophila melanogaster*

**DOI:** 10.1016/j.jgg.2017.06.003

**Published:** 2017-10-20

**Authors:** Dwayne J. Byrne, Mark J. Harmon, Jeremy C. Simpson, Craig Blackstone, Niamh C. O'Sullivan

**Affiliations:** a UCD School of Biomolecular and Biomedical Science, UCD Conway Institute, University College Dublin, Dublin 4, Ireland; b UCD School of Biology and Environmental Science, UCD Conway Institute of Biomolecular and Biomedical Research, University College Dublin, Dublin 4, Ireland; c Cell Biology Section, Neurogenetics Branch, National Institute of Neurological Disorders and Stroke, National Institutes of Health, Bethesda, MD 20892, USA

**Keywords:** Proteasome, TDP-43 accumulation, Neurodegeneration, *Drosophila*, ALS, amyotrophic lateral sclerosis, IBMPDF, inclusion body myopathy with Paget's disease of the bone and frontotemporal dementia, NMJ, neuromuscular junction, Npl4, nuclear protein localization homolog 4, TBPH, TAR DNA-binding protein-43 homolog, Ufd1, ubiquitin fusion degradation 1, UPS, ubiquitin proteasome system, VCP, valosin-containing protein

## Abstract

The VCP-Ufd1-Npl4 complex regulates proteasomal processing within cells by delivering ubiquitinated proteins to the proteasome for degradation. Mutations in VCP are associated with two neurodegenerative diseases, amyotrophic lateral sclerosis (ALS) and inclusion body myopathy with Paget's disease of the bone and frontotemporal dementia (IBMPFD), and extensive study has revealed crucial functions of VCP within neurons. By contrast, little is known about the functions of Npl4 or Ufd1 *in vivo*. Using neuronal-specific knockdown of *Npl4* or *Ufd1* in *Drosophila melanogaster*, we infer that Npl4 contributes to microtubule organization within developing motor neurons. Moreover, *Npl4* RNAi flies present with neurodegenerative phenotypes including progressive locomotor deficits, reduced lifespan and increased accumulation of TAR DNA-binding protein-43 homolog (TBPH). Knockdown, but not overexpression, of *TBPH* also exacerbates *Npl4* RNAi-associated adult-onset neurodegenerative phenotypes. In contrast, we find that neuronal knockdown of Ufd1 has little effect on neuromuscular junction (NMJ) organization, TBPH accumulation or adult behaviour. These findings suggest the differing neuronal functions of Npl4 and Ufd1 *in vivo*.

## 1 Introduction

The ubiquitin proteasome system (UPS) is the primary protein processing system of the cell which ensures that misfolded, ubiquitin-tagged proteins are translocated to the proteasome where they are degraded. Central to this process is the action of the highly conserved chaperone complex VCP-Ufd1-Npl4, made up of the hexameric VCP (valosin-containing protein) bound to Ufd1 (ubiquitin fusion degradation 1) and Npl4 (nuclear protein localization homolog 4). Misfolded proteins are identified and polyubiquitinated by the action of ubiquitin activating (E1), conjugating (E2), and ligase (E3) enzymes (Pickart, 2001). Subsequently, the VCP-Ufd1-Npl4 complex binds to the ubiquitin-tagged proteins, extracts them out and away from non-ubiquitinated partners, and delivers them to the proteasome for processing (Ye et al., 2001). All components of the VCP-Ufd1-Npl4 complex are highly conserved across species and loss of any one of these proteins impairs UPS function, triggering accumulation of ubiquitinated proteins in yeast, *Drosophila* and mammalian systems (Dai and Li, 2001, Ye et al., 2001, Wojcik et al., 2004, Beskow et al., 2009).

Of the proteins in the VCP-Ufd1-Npl4 complex, VCP has been extensively studied and has been shown to have multifaceted roles in various cellular pathways. Specificity of VCP function is determined by its association with different protein co-factors such that VCP binding to p37 functions in Golgi/ER biogenesis (Uchiyama et al., 2006), VCP binding to p47 functions in membrane fusion (Roy et al., 2000), VCP binding to UBXD1 functions in endosomal sorting (Ritz et al., 2011), and VCP binding to Npl4-Ufd1 regulates UPS functions (Ye et al., 2003). Within post-mitotic neurons, regulation of the UPS by VCP is critically important. Mutations in VCP are causative of two fatal proteinopathies: amyotrophic lateral sclerosis (ALS) and inclusion body myopathy with Paget's disease of the bone and frontotemporal dementia (IBMPFD) (Watts et al., 2004, Johnson et al., 2010, Abramzon et al., 2012). Both of these degenerative diseases are characterised by cytoplasmic aggregates containing ubiquitinated and disease-causing mutant proteins within neurons and glia, most frequently the RNA-binding protein TAR-DNA binding protein 43 (TDP-43) (Neumann et al., 2006). Moreover, neurodegeneration caused by pathogenic VCP mutations is exacerbated by cytoplasmic mislocalization of TDP-43 in *Drosophila* models of IMBPFD (Ritson et al., 2010). Together, these results highlight an important role for VCP in neuronal maintenance *in vivo*.

In contrast, relatively little is known about the functions of the VCP co-factors Npl4 and Ufd1, and specific roles for these proteins in neuronal function and dysfunction have not been investigated. Npl4 and Ufd1 undergo bipartite binding to VCP, via the UBXL domain on Npl4 and the SHP1 domain on Ufd1, to extract ubiquitinated polypeptides for degradation by the proteasome (Ye et al., 2001, Bruderer et al., 2004, Hanzelmann and Schindelin, 2016). The substrates of VCP-Ufd1-Npl4-mediated UPS include transcription factors (Zhang et al., 2015), cell cycle regulators (Cao et al., 2003), mitochondrial targets for the clearance of damaged mitochondria (Kim et al., 2013) and aggregate prone proteins (Ballar et al., 2011). Though little work has investigated independent roles for Ufd1 and Npl4, some *in vitro* studies have revealed different requirements for Ufd1 and Npl4 in the UPS degradation of substrates of certain ubiquitin E3 ligases (Ballar et al., 2006, Ballar et al., 2011, Cao et al., 2007). For example, in yeast, the action of Npl4, but not Ufd1, is required to degrade the primary pathogenic form of the cystic fibrosis transmembrane conductance regulator (CFTR) ΔF508 (Ballar et al., 2011). Interestingly, CFTRΔF508 forms cytoplasmic aggregates in the affected tissues of cystic fibrosis patients, resembling TDP-43 aggregates in ALS and IMBPFD patients, though it is not yet known how this contributes to disease pathogenesis (Du et al., 2015).

In this study, we set out to better understand the role of the VCP co-factors Npl4 and Ufd1 in neuronal function *in vivo*. We have generated *Npl4* and *Ufd1* reduced expression *Drosophila* by targeted knockdown of these genes. We find that both Ufd1 and Npl4 are required for neuronal organization and function, though knockdown of *Npl4* results in much more severe phenotypes. Furthermore, knockdown of *Npl4*, but not *Ufd1*, is associated with increased accumulation of TBPH, the *Drosophila* homolog of TDP-43, and modulates loss of TBPH-associated neurodegenerative phenotypes. These findings suggest a specific role for Npl4-dependent proteasomal function in neurons and provide a novel model to decipher the pathogenic effect of proteasomal inhibition within neurons *in vivo*.

## 2 Results

### 2.1 Npl4 and Ufd1 have roles in the development and maintenance of neuronal function

All three components of the VCP-Ufd1-Npl4 complex have well conserved *Drosophila* homologs: Ter94, Ufd1-like and Npl4, that share 85%, 56% and 48% protein sequence identity with human sequences, respectively. *Drosophila* lines containing *Ufd1* and *Npl4* RNAi insertions are available from the Vienna *Drosophila* RNAi centre (Dietzl et al., 2007; www.VDRC.at). Expression of *Npl4* RNAi or *Ufd1* RNAi under the control of the ubiquitous driver *da-GAL4* results in similarly robust knockdown of gene expression (Figs. 1A and S1) and lethality during late larval or pupal stages of development. This is consistent with the essential functions of the VCP-Ufd1-Npl4 complex during development, with loss of Ufd1 or Npl4 causing developmental lethality (Mouysset et al., 2006). The targeted knockdown is specific for each gene such that *Npl4* RNAi has no significant effect on Ufd1 expression and *vice versa*. Furthermore, neither *Npl4* nor *Ufd1* RNAi lines resulted in altered VCP expression levels. RNAi lines from the KK library were used throughout this study as the *Npl4* RNAi and *Ufd1* RNAi lines from the GD library failed to strongly modify expression of the target genes, with *Drosophila* surviving all stages of development. The 60100 *w*^*1118*^ control stock, which is the genetic background for all KK RNAi lines, was used as the control line throughout this study.

To study whether Npl4 or Ufd1 has neuronal-specific functions, we investigated the effect of neuronal-specific knockdown of these genes by crossing RNAi lines to the pan-neuronal driver *nSyb-GAL4*. Two *Drosophila* lines with neuronal loss of Npl4 or Ufd1 are viable and appear healthy but has discernible locomotor deficits compared to controls at the larval L3 stage, with a more severe locomotor deficit detectable in *Npl4* RNAi larvae than *Ufd1* RNAi larvae (Fig. 1B). In adult *Drosophila*, neuronal knockdown of *Npl4* or *Ufd1* causes an early onset progressive decline in locomotor activity relative to controls, from 9 to 24 days post-eclosion in *Npl4* RNAi flies and from 30 days post-eclosion in *Ufd1* RNAi flies (Fig. 1C). Lifespan assays of these flies reveal that *Npl4* RNAi flies have significantly impaired survival compared to controls, reflecting a degenerative phenotype resulting from neuronal loss of Npl4 (Fig. 1D; Tables S1 and S2). In contrast, survival in *Ufd1* RNAi flies is unchanged from that of controls.

**Fig. 1:**
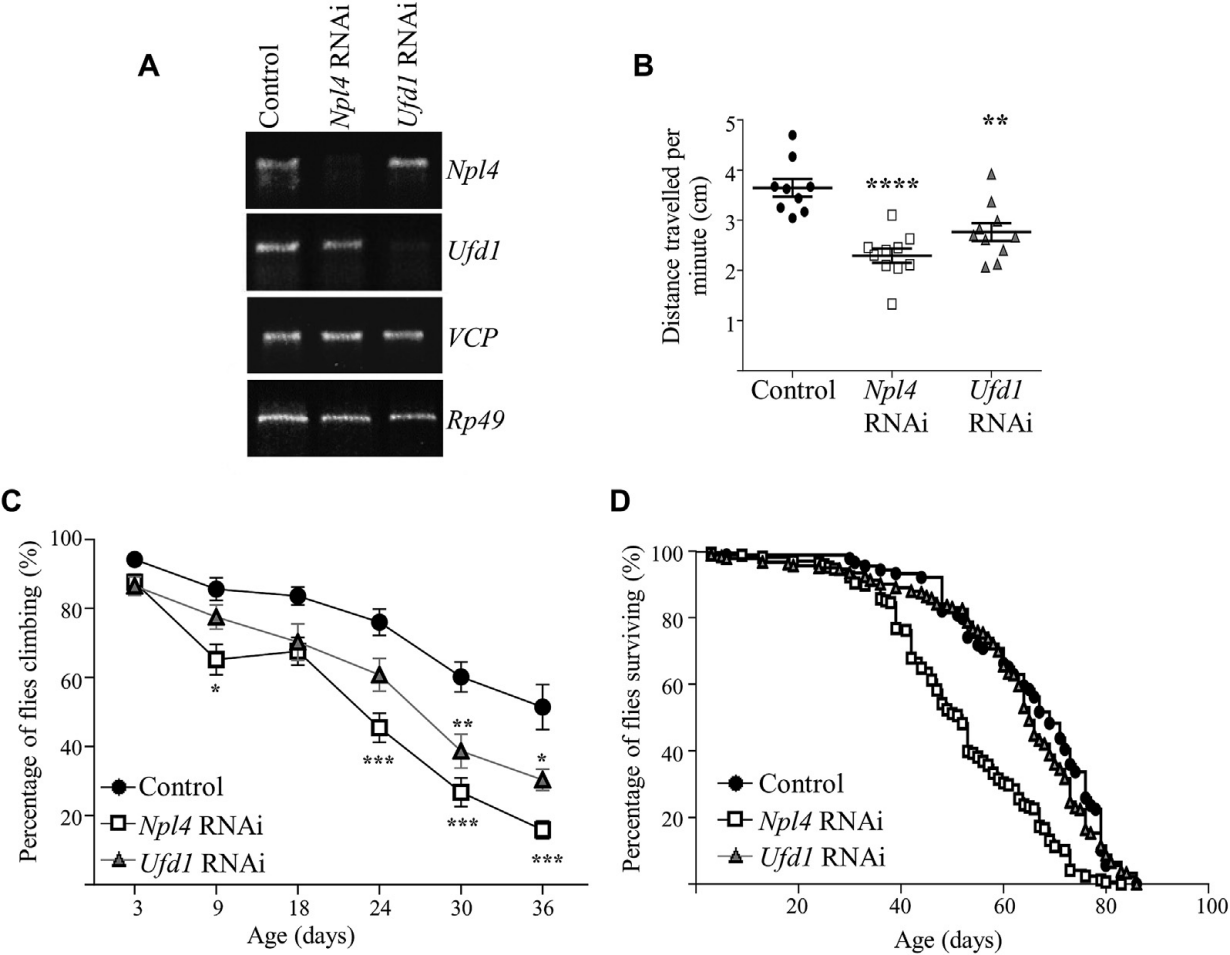
Neuronal knockdown of *Drosophila Npl4* or *Ufd1* causes progressive degeneration. **A**: Targeted expression of RNAi lines successfully knocked down *Npl4* or *Ufd1* expression as evidenced by PCR amplification of *Npl4*, *Ufd1* and *Rp49* cDNA from progeny of *da-GAL4* crossed to either 60100 *w*^*1118*^ (control), *Npl4* RNAi or *Ufd1* RNAi flies. **B** and **C**: Neuronal-specific loss of *Npl4* or *Ufd1* (generated using *nSyb-GAL4*) caused locomotor deficits in larvae (**B**, values significantly different from control were determined by one-way ANOVA and Tukey's post-tests; ****P < 0.0001, **P < 0.01; *n* = 9–10 experiments; 10 larvae per experiment) and adult flies (**C**, values significantly different from control were determined by two-way ANOVA and Bonferroni's post-tests; *, *P* < 0.05; **, *P* < 0.01; ***, *P* < 0.005; *n* = 11–19 experiments; 10 flies per experiment) compared to controls. **D**: Neuronal-specific loss of *Npl4*, but not *Ufd1*, caused decreased lifespan compared to controls (*P* < 0.0001 by log-rank test; *n* = 90–180 flies).

VCP interacts with Npl4 and Ufd1 to mediate the proteasomal clearance of ubiquitinated proteins as part of the UPS. Loss of either cofactor results in a failure of this clearance and an accumulation of ubiquitinated proteins, which is not seen upon loss of other VCP cofactors (Wojcik et al., 2004). Accumulation of unfolded or misfolded proteins in the endoplasmic reticulum (ER) activates the ER stress response, a major branch of which is the IRE1/Xbp1 pathway (Sone et al., 2013). In this pathway, *Xbp1* mRNA is spliced by activated IRE1 resulting in the translation of the active transcription factor Xbp1(s) that induces the expression of downstream target genes including quality control proteins (Yamamoto et al., 2007). In the absence of ER stress, *Xbp1* mRNA is not spliced and the resulting Xbp1(u) cannot function to activate transcription of these genes. The presence of Xbp1(s) can therefore be used as a read-out of increased ER stress. To determine whether there was an upregulation of ER stress in response to loss of Npl4 or Ufd1, we used an antibody which detects both Xbp1(s) and Xbp1(u). Loss of either Npl4 or Ufd1 results in expression of activated Xbp1(s) which is not detected in control larvae (Fig. 2). Together, these results demonstrate that knockdown of *Npl4* or *Ufd1* induces ER stress and causes progressive neurodegeneration.

**Fig. 2:**
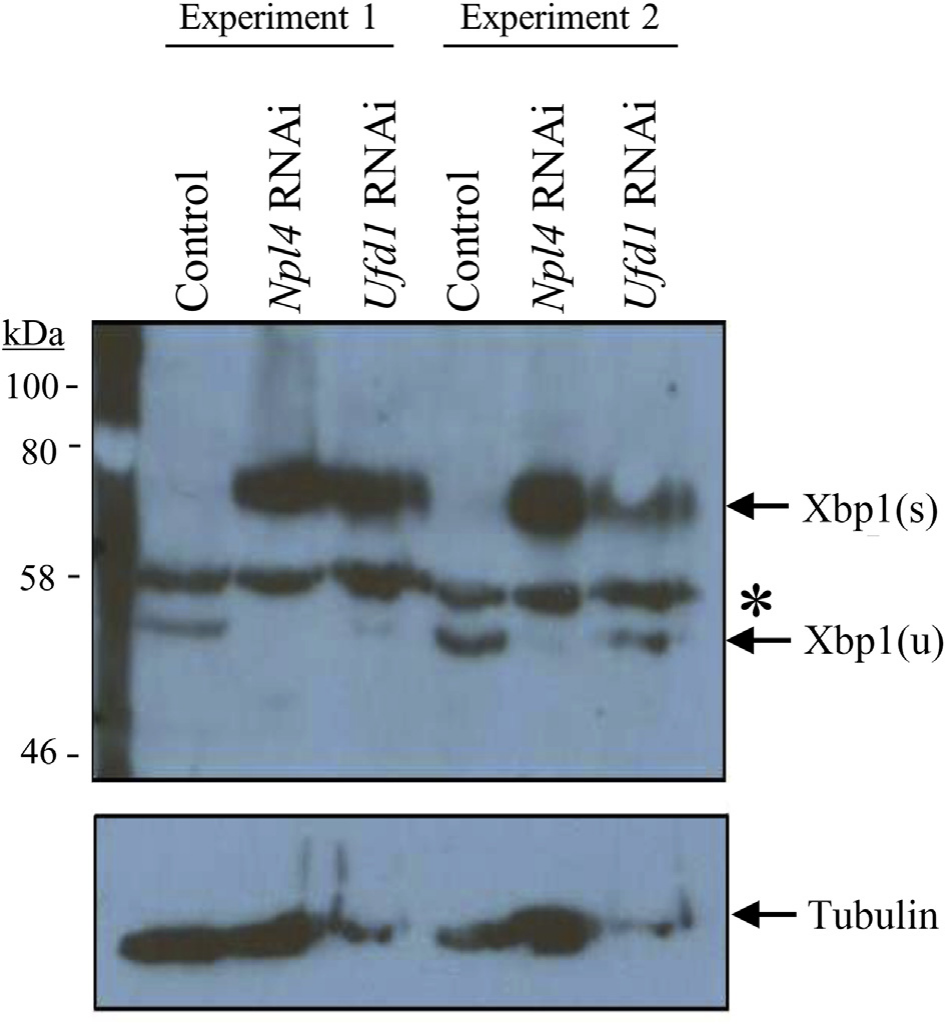
Knockdown of *Ufd1* and *Npl4* induces splicing of ER stress factor, Xbp1. Western blot analysis of protein samples purified from larval progeny of *da-GAL4* crossed to either control, *Npl4* RNAi or *Ufd1* RNAi flies from two independent experiments is shown. Knockdown of *Npl4* or *Ufd1* resulted in the production of activated Xbp1(s) and the reduction of inactive Xbp1(u). The asterisk denotes a non-specific band. The nitrocellulose membrane was stripped and reprobed with antibody against α-tubulin.

### 2.2 Npl4 is required for normal microtubule and neuromuscular junction organization

Given the locomotor phenotypes evident in *Npl4* RNAi *Drosophila*, and to a lesser extent in *Ufd1* RNAi *Drosophila*, we investigated the effect of knockdown of *Npl4* or *Ufd1* on neuronal structure. Synaptic morphology was examined in control and neuronal loss of Npl4 or Ufd1 stage L3 larvae by staining neuromuscular junctions (NMJs) with anti-HRP antibodies, permitting visualization of the presynaptic membrane. Image analysis of *Npl4* RNAi larvae revealed a significantly elongated NMJ morphology indicated by increased branch length at muscle 6/7 in abdominal segment A3 compared to controls (Fig. 3A and B). This phenotype may reflect defective neuronal pruning, and is reminiscent of the long peripheral neuronal dendritic arborisations evident in VCP mutants (Rumpf et al., 2014). In contrast, *Ufd1* RNAi NMJ branch length was unchanged from control.

**Fig. 3:**
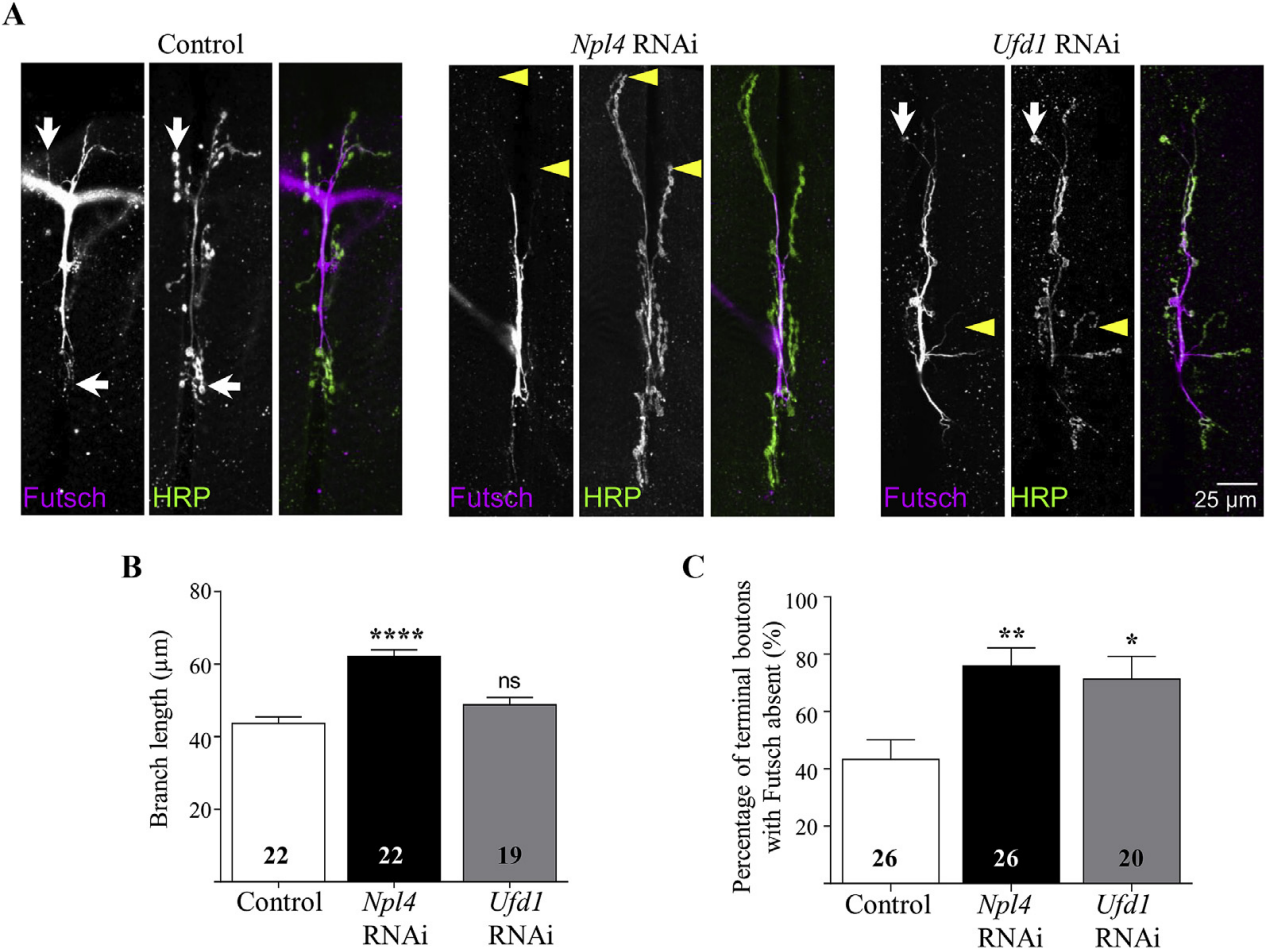
Neuromuscular junction organization in neuronal knockdown *Npl4* or *Ufd1* larvae. **A**: Confocal images showing NMJs on muscles 6/7 of L3 larvae stained for the microtubule marker Futsch (magenta) and the neuronal membrane marker HRP (green). Larvae are progeny of *nSyb-GAL4* crossed to either control, *Npl4* RNAi or *Ufd1* RNAi flies. **B**: Quantification of NMJ branch length per NMJ. **C**: Graph represents percentage of branches in which the terminal boutons lack of Futsch staining. Knockdown of *Npl4* or *Ufd1* impaired microtubule organization as evidenced by the absence of Futsch staining in the most distal terminal boutons (yellow arrowheads in **A**) compared to controls (arrows in **A**). Data are expressed as mean ± SEM (*n* = 20–26 larvae from three independent experiments with *n* for each genotype indicated on graphs), and values significantly different from control were determined by one-way ANOVA and Dunnett's post-tests (*, *P* < 0.05; **, *P* < 0.01; ****, *P* < 0.0001; ns, *P* > 0.05).

These NMJ morphological defects prompted us to investigate the organization of the neuronal cytoskeleton within presynaptic terminals by staining with antibodies to the microtubule (MT)-associated protein Futsch (the *Drosophila* homolog of vertebrate MAP1B) (Hummel et al., 2000). MT Futsch staining extends the length of neurons and is normally present in the distal terminal boutons of wild-type controls (Fig. 3A, arrows). However, Futsch staining is notably absent from the terminal boutons of *Npl4* RNAi and many *Ufd1* RNAi, and NMJ branches (Fig. 3A, arrowheads). Quantification reveals a significant increase in the proportion of branches in which the terminal bouton is lacking Futsch staining in *Npl4* and *Ufd1* RNAi NMJs at muscle 6/7 in anterior segment A3 (Fig. 3C). Futsch staining is also reduced within axon bundles of *Npl4* RNAi neurons compared to controls (Fig. S2A and B). Comparable morphological and Futsch staining results were obtained when NMJs at muscle 6/7 and axon bundles in posterior segment A7 were analyzed (Fig. S3A and B), indicating that there is no length-dependent effect due to loss of Npl4 or Ufd1 from neurons as has been identified in other models of neurodegeneration (Fowler and O'Sullivan, 2016). *Futsch* mRNA expression levels were unaffected by loss of Npl4 or Ufd1, suggesting that the observed disruption is not caused by altered *Futsch* gene expression (Fig. S2C).

We investigated whether other intracellular compartments were disrupted by loss of Npl4 or Ufd1. Axonal staining of the smooth ER marker Rtnl1:YFP is unchanged in *Npl4* RNAi or *Ufd1* RNAi motor neurons compared to controls (Fig. S2A). Furthermore, analysis of mitochondrial intensity, area and circularity revealed no change in mitochondrial organization in the axons of these larvae (Fig. S4). These findings suggest that the disruption of MT organization in *Npl4* RNAi motor neurons *in vivo* occurs independently of gross, widespread defects in intracellular compartments.

### 2.3 Knockdown of Npl4 increases neuronal mislocalization of TBPH

**Fig. 4:**
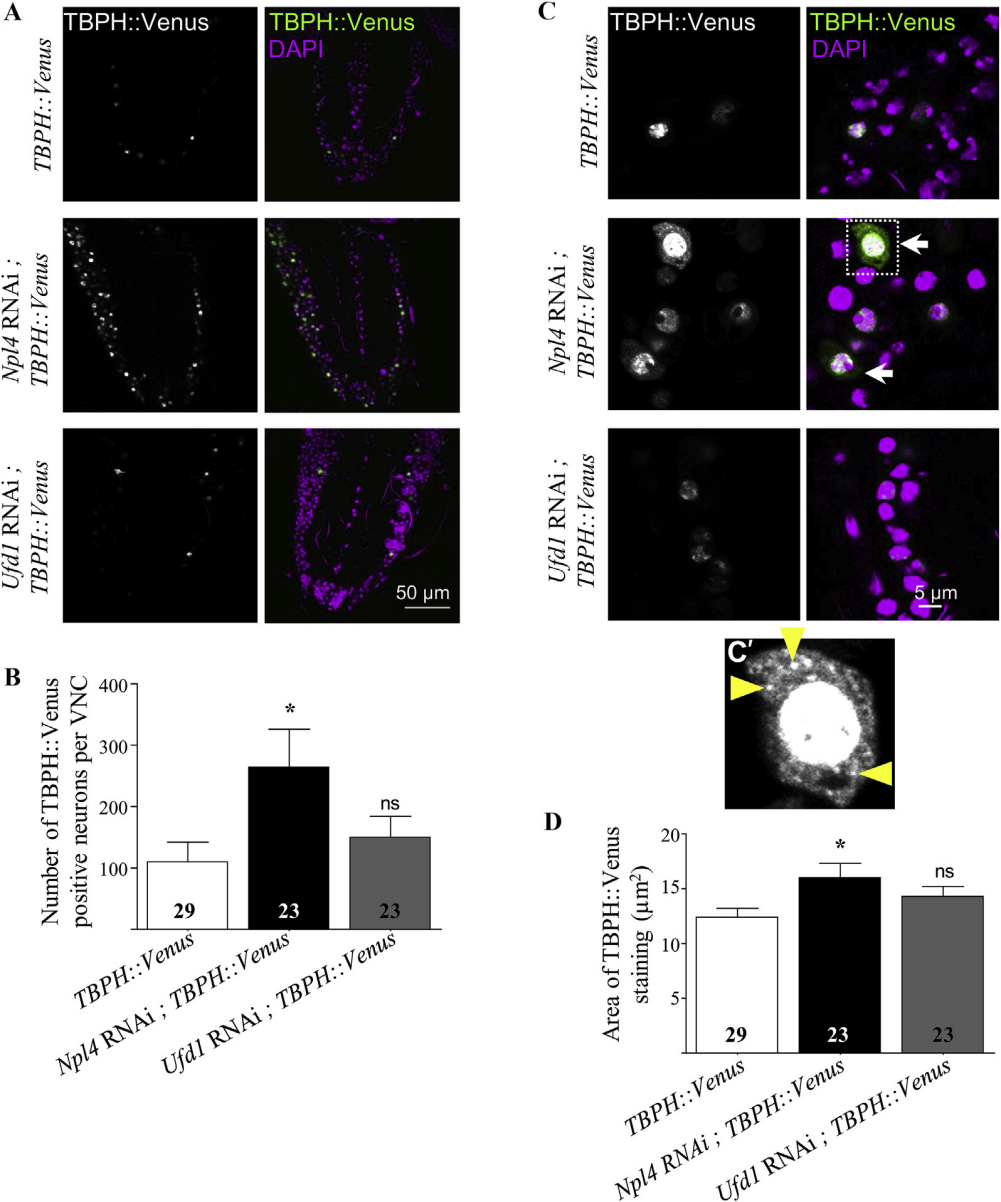
Neuronal knockdown of *Npl4* increases TBPH accumulation. **A**: Confocal sections of larval ventral nerve cord (VNC) showing Venus-tagged TBPH (green) and DAPI (magenta) staining in control, *Npl4* RNAi or *Ufd1* RNAi neurons (generated using *nSyb-GAL4*). **B**: Graph shows quantification of the number of TBPH::Venus positive neurons throughout the VNC in each genotype. **C**: Knockdown of *Npl4* caused increased mislocalization of TBPH to the cytoplasm compared to control (arrows). Inset (**C′**) shows cytoplasmic accumulations of TBPH detectable in a subset of *Npl4* RNAi neurons (yellow arrowheads). **D**: Graph shows quantification of area of neuronal TBPH::Venus positive staining in each genotype. All data are expressed as mean ± SEM (*n* = 23–29 larvae from 5 independent experiments), and values significantly different from control were determined by one-way ANOVA and Dunnett's post-tests (*, *P* < 0.05; ns, *P* > 0.05).

Disease-causing mutations in VCP cause cytoplasmic redistribution of the ALS-associated protein TDP-43 (TBPH in *Drosophila)* (Ritson et al., 2010). We therefore examined the effect of knockdown of *Npl4* or *Ufd1* on TBPH distribution in neurons. Venus-tagged TBPH (Wang et al., 2011) was expressed in neurons under the control of *nSyb-GAL4* in control, *Npl4* RNAi or *Ufd1* RNAi larvae. Entire ventral nerve cords (VNCs) were imaged at 5 μm intervals and TBPH::Venus staining was quantified. In VNCs of control and *Ufd1* RNAi larvae, a subset of neurons showed intense TBPH::Venus staining, identified as positively stained when analyzed in ImageJ. Knockdown of Npl4 caused a striking increase in the number of TBPH::Venus positive neurons (Fig. 4A and B). This suggests that loss of Npl4 renders neurons more susceptible to accumulation of this aggregate-prone protein, possibly through impaired proteasomal processing.

TDP-43 mislocalization (to the cytoplasm from the nucleus) as well as cytoplasmic and nuclear aggregates of TDP-43 are common features in ALS/FTD models and in neurons from ALS/FTD patients (Mouysset et al., 2006, Neumann, 2009). TBPH::Venus expressed in control and *Ufd1* RNAi neurons overlapped with DAPI staining and therefore localized to nuclei (Fig. 4C), consistent with previous findings (Alami et al., 2014). In contrast, loss of Npl4 caused increased localization of TBPH in the cytoplasm of neurons (Fig. 4C, arrows) with cytoplasmic aggregates of TBPH evident in a subset of *Npl4* RNAi neurons (Fig. 4C′, arrowheads). Analysis of all TBPH::Venus-positive cells revealed that loss of Npl4 results in a larger area of staining of TBPH::Venus, confirming cytoplasmic mislocalization of TBPH (Fig. 4D).

### 2.4 TBPH knockdown modulates Npl4 RNAi- and Ufd1 RNAi-associated neurodegenerative phenotypes

**Fig. 5:**
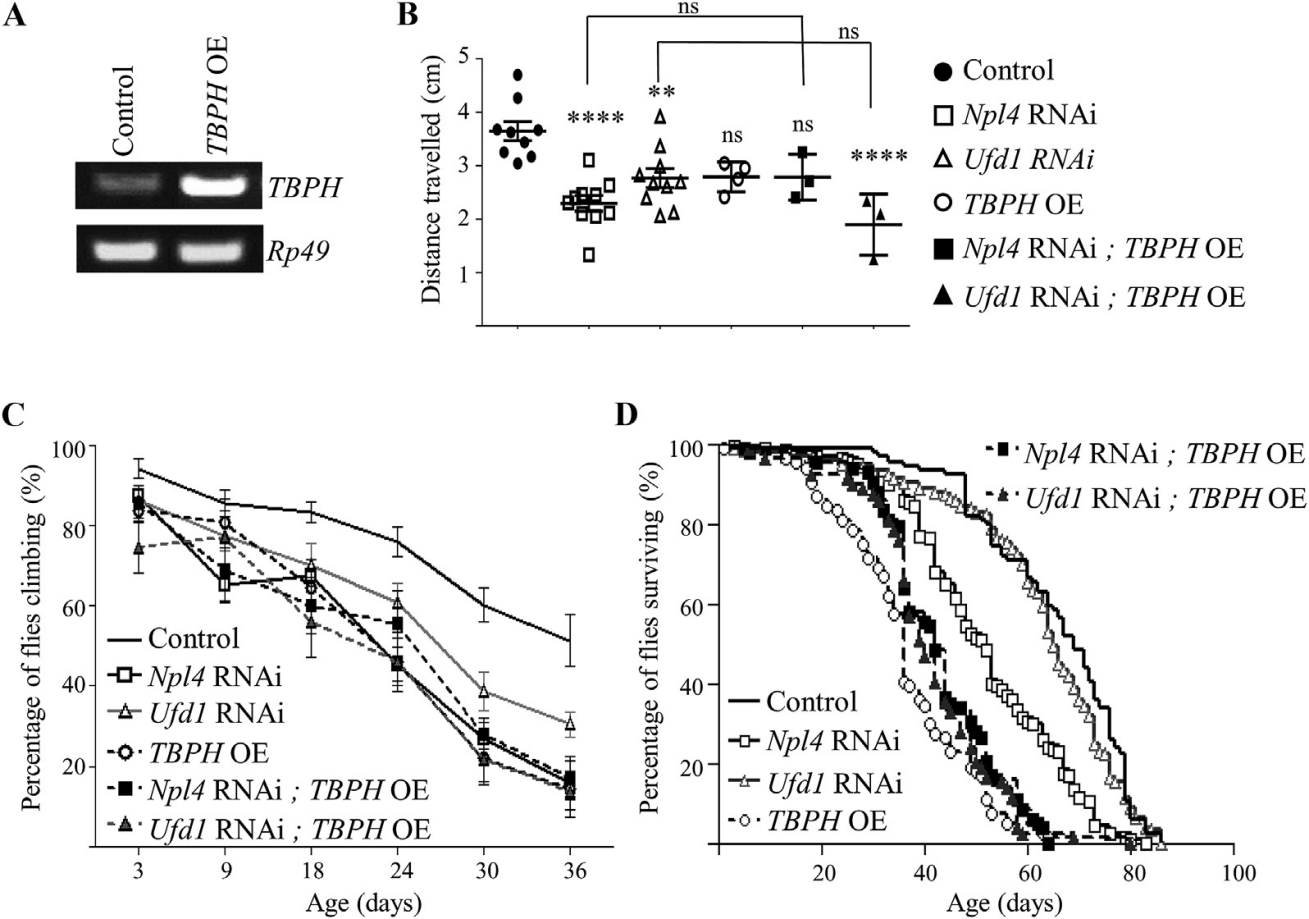
Overexpression of *TBPH* does not modify neurodegeneration associated with knockdown of *Npl4* or *Ufd1*. **A**: PCR validation of *TBPH* overexpression (OE) in *UAS-TBPH::Venus* larvae compared to control larvae. **B** and **C**: Neuronal-specific OE of *TBPH* did not significantly modify locomotor deficits caused by knockdown of *Npl4* or *Ufd1* (generated using *nSyb-GAL4*) in either larvae (**B**, *P* values determined by one-way ANOVA and Tukey's post-tests; *n* = 3–10 experiments, 10 larvae from per experiment; ns *P* > 0.05, **P < 0.01, ****P < 0.0001) or adult flies (**C**, *n* = 8–18 experiments, 10 flies per experiment). **D**: Neuronal OE of *TBPH* did not significantly modify the lifespan of these flies (*P* > 0.05 by log-rank tests; *n* = 84–180 flies).

To determine whether this increased TPBH accumulation caused by expression of TPBH::Venus in *Npl4* knockdown neurons has any physiological consequences, we looked for effects on the locomotor or survival deficits caused by loss of Npl4 or Ufd1 alone. Neuronal overexpression (OE) of *TBPH* (Fig. 5A) causes impaired locomotion in adult flies and reduction of lifespan consistent with previous findings (Fig. 5C and D; Tables S1‒S4) (Diaper et al., 2013). The combined effect of neuronal knockdown of *Npl4* and OE of *TBPH* on adult locomotion and survival is intermediate to the effect of either *Npl4* RNAi or *TBPH* OE alone (Fig. 5B–D; Table S1‒S4). Similarly, the combined effect of *Ufd1* RNAi and *TBPH* OE on adult locomotion and survival is similar to the effect of *TBPH* OE alone (Fig. 5C and D; Table S1‒S4). This argues against an additive effect of *TBPH* OE in either *Npl4* RNAi or *Ufd1* RNAi *Drosophila*.

**Fig. 6:**
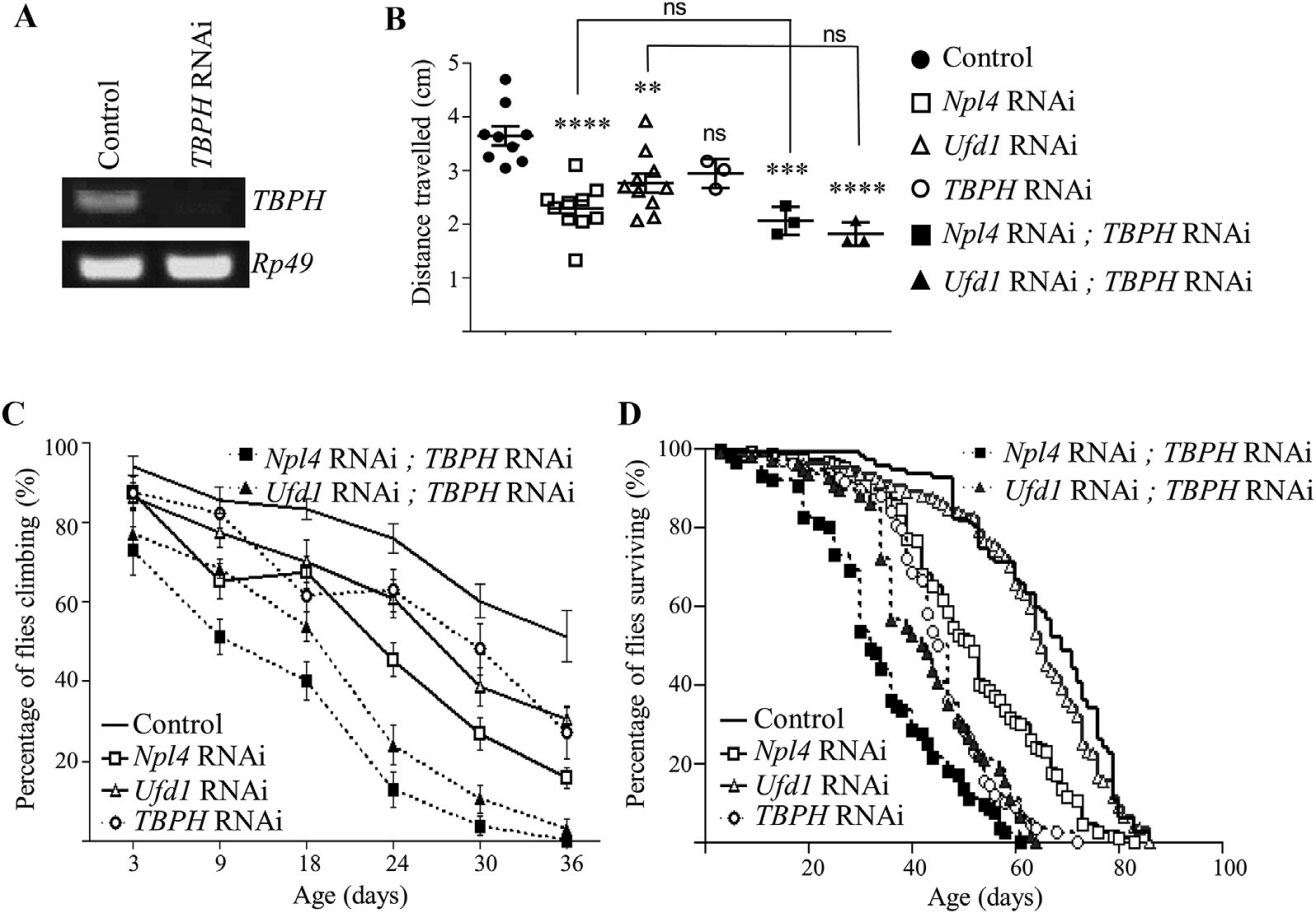
Knockdown of *TBPH* exacerbates neurodegeneration associated with knockdown of *Npl4* or *Ufd1*. **A**: PCR validation of *TBPH* knockdown in *TBPH RNAi* larvae compared to control larvae. **B** and **C**: Locomotor deficits caused by neuronal specific loss of *Npl4* or *Ufd1* (generated using *nSyb-GAL4*) were exacerbated by *TBPH* knockdown in adult flies only. Graphs show quantification of locomotor scores in larvae (**B**, *P* values determined by one-way ANOVA and Tukey's post-tests; *n* = 3–10 experiments, 10 larvae per experiment; **, *P* < 0.01; ***, *P* < 0.001; ****, *P* < 0.0001; ns, *P* > 0.05) or adult flies (**C**, *P* values determined by two-way ANOVA and Bonferroni's post-tests; *n* = 8–18 experiments, 10 flies per experiment). **D**: Loss of *TBPH* significantly shortened the lifespan of *Npl4* knockdown flies compared to loss of either *Npl4* or *TBPH* alone (*P* < 0.0001 by log-rank tests; *n* = 84–180 flies). Note that control, *Npl4* RNAi and *Ufd1* RNAi values are the same as those used in Fig. 5.

Previous reports have suggested that in *Drosophila*, TBPH aggregates may not themselves confer neurotoxicity but instead loss-of-function phenotypes may underlie TBPH-dysfunction in *Drosophila* (Diaper et al., 2013, Cragnaz et al., 2014). TBPH is required during development and few *TBPH* knockout *Drosophila* survive to adulthood (Feiguin et al., 2009, Diaper et al., 2013). We therefore investigated the effect of loss of TBPH on *Npl4* RNAi and *Ufd1* RNAi *Drosophila* using *TBPH* RNAi (Diaper et al., 2013) under the control of *nSyb-GAL4*. While neuronal *TBPH* RNAi (Fig. 6A) does not significantly disrupt larval locomotion (Fig. 6B), adult climbing and survival are reduced in *TBPH* RNAi adults (Fig. 6C and D; Table S1‒S4). In contrast to the findings with *TBPH* OE, *TBPH* RNAi exacerbates neurodegenerative phenotypes caused by knockdown of *Npl4* and both adult climbing and survival were significantly reduced in *Npl4* RNAi and *TBPH* RNAi double knockdown *Drosophila* compared to either *Npl4* RNAi or *TBPH* RNAi alone (Fig. 6C and D; Tables S1 and S2). Adult locomotion, but not larval locomotion or survival, was also significantly impaired in *Ufd1* RNAi and *TBPH* RNAi double knockdown *Drosophila* compared to either *Ufd1* RNAi or *TBPH* RNAi alone (Fig. 6B–D; Table S1‒S4). These results suggest that in *Drosophila*, age-dependent neurodegeneration associated with Npl4 and Ufd1 knockdown is more sensitive to reduced TPBH expression than *TBPH* OE.

## 3 Discussion

The VCP-Ufd1-Npl4 complex is critically required in all cells to regulate protein processing and degradation via UPS. However, while VCP has been studied extensively in cell and animal systems, the co-factors Npl4 and Ufd1 remain relatively poorly examined (Roy et al., 2000, Ye et al., 2003, Watts et al., 2004, Uchiyama et al., 2006, Johnson et al., 2010, Ritson et al., 2010, Ritz et al., 2011, Abramzon et al., 2012, O'Sullivan et al., 2013). Here, we have generated *Drosophila* in which neuronal expression of Npl4 and Ufd1 has been knocked down to identify specific roles for these proteins in post-mitotic neurons *in vivo*.

An important finding of this study is that comparable knockdown of *Npl4* and *Ufd1* from *Drosophila* neurons causes overlapping yet distinct neurological consequences. In fact, we have found that knockdown of *Npl4* results in more severe neurodevelopmental and neurodegenerative behavioural phenotypes than knockdown of *Ufd1*. These differences in neuronal phenotypes occur despite the similarly robust reduction of either *Npl4* or *Ufd1* gene expression in their respective knockdown lines and the induction of the ER stress response, as determined by Xbp1 splicing, in both lines. Instead, we have found evidence that these behavioural phenotypes may be brought about by defects in neuronal microtubule network formation and aberrant NMJ organization which are particularly evident in *Npl4* knockdown *Drosophila*. Loss of VCP has recently been shown to regulate dendritic spine density in cultured mammalian neurons, via a p47-and protein synthesis-dependent pathway and knockdown of *Ufd1* did not alter dendritic spine density (Shih and Hsueh, 2016), consistent with the findings presented here. Unfortunately, knockdown of *Npl4* was not investigated, therefore it is not currently known whether Npl4 functions along with VCP in mammalian neuronal dendritic spine formation.

Mutations in the gene encoding VCP cause cytoplasmic aggregation of TDP-43/TBPH in animal models of IMBPFD (Ritson et al., 2010) and in the muscles of IMBPFD patients (Weihl et al., 2008). We now provide evidence that reduced expression of the VCP co-factor Npl4 is sufficient to induce TBPH aggregation within a subset of neuronal cell bodies. These findings may be explained by a model whereby VCP-Npl4, and perhaps to a lesser extent VCP-Ufd1-Npl4, are required for the correct processing of TDP-43. While this function cannot be directly linked to the degenerative phenotypes observed in the *Npl4* RNAi *Drosophila*, we have found that *Npl4* knockdown exacerbates neurodegeneration associated with loss of TBPH. This finding contrasts with previous work showing that *TDP-43* RNAi partially rescues neurotoxicity associated with overexpression of mutant forms of VCP, while overexpression of TDP-43 exacerbates neurotoxicity in the model (Ritson et al., 2010). Nonetheless, our results point to a role for Npl4 in neuronal maintenance which may be important when considering therapeutic strategies to increase the clearance of neurotoxic TDP-43 in IMBPFD or ALS (Azuma et al., 2014).

An important question that remains to be addressed is the mechanism by which Npl4 and Ufd1 have distinct functions in neurons. One possible explanation could be that functional VCP-Npl4 complexes form even in the absence of Ufd1. 3D electron cryomicroscopic reconstructions of the VCP-Ufd-Npl4 complex reveal that Npl4 mediates the interaction between Npl4/Ufd1 and VCP by binding to the N-domain of VCP, while binding sites on Ufd1 allow for higher affinity binding through the large degree of conformational flexibility in the VCP-Ufd1-Npl4 complex (Bebeacua et al., 2012). Therefore, VCP-Npl4 complexes may form in cells independent of Ufd1. A second, not mutually exclusive, explanation is that there exist distinct substrates for Npl4-and Ufd1-containing complexes. The E3 ubiquitin ligase gp78 has been shown to mediate degradation of its substrates via the VCP-Npl4 complex, independently of Ufd1 (Ballar et al., 2011). gp78 is highly involved in the degradation of neurodegenerative disease proteins and its substrates are known to include the aggregate-prone proteins SOD1, Ataxin-3, mutant Huntingtin and CFTRΔF508 (Yang et al., 2007, Yang et al., 2010, Ying et al., 2009, Ballar et al., 2011). Given the findings presented here, it is interesting to speculate that VCP-Npl4 complexes may function within neurons *in vivo* to regulate the degradation of aggregate-prone proteins such as TDP-43.

## 4 Materials and methods

### 4.1 Fly stocks

All fly stocks were maintained as described previously (Fowler and O'Sullivan, 2016). Briefly, flies were maintained at 25°C with 12 h:12 h light:dark and were transferred to fresh cornmeal media containing vials every 2–3 days. For knockdown experiments, the following fly lines were used: *UAS-Npl4-RNAi* line 109309 (construct KK102533); *UAS-Ufd1-RNAi* line 104713 (construct KK108134); the 60100 *w*^*1118*^ control stock (all obtained for the Vienna *Drosophila* RNAi Centre, www.vdrc.at) (Dietzl et al., 2007); *UAS-TBPH::Venus* (generated by Brian McCabe) (Wang et al., 2011); *UAS-TBPH-RNAi* (generated by Frank Hirth) (Diaper et al., 2013). UAS lines were crossed either to *da-GAL4* (Perrin et al., 2003), *nSyb-GAL4* (Bushey et al., 2009) or *OK6-GAL4* (Aberle et al., 2002) as indicated in the text.

### 4.2 Behavioural assays

All behavioural assays were conducted on progeny of *nSyb-Gal4* crossed to 60100 *w*^*118*^ (control) or RNAi flies. Larval locomotor abilities were tested by placing larvae on the centre square of a grid on a 9 cm Petri dish, marked with a 1 cm^2^ grid, containing 2% agarose (poured and set prior to testing) and counting the number of grid lines crossed in 1 min. 10 larvae per genotype per day were investigated. A minimum of 3 independent experiments, testing 2–3 vials of 10 male flies each, were used to examine adult locomotion. Flies were maintained at 25°C and assessed on designated days post-eclosion. The number of flies climbing to the top of a vertical glass vial (10 cm length, 2.5 cm diameter) over 15 s was determined. Survival assays consisted of taking a daily mortality score on male flies from climbing assays.

### 4.3 Semi-quantitative PCR

PCR was performed on cDNA produced from RNA isolated and purified from 10 larvae collected in TRIzol reagent (Invitrogen, Paisley, UK) as previously described (Fowler and O'Sullivan, 2016). Primers used were: Rp49-F: 5ʹ-CCGACCACGTTACAAGAACTCTC; Rp49-R: CGCTTCAAGGGACAGTATCTGA; Npl4-F: 5ʹ-GCGAACGTGATAGCAAGCTG-3ʹ; Npl4-R: 5ʹ-TACTCGCATCGTCCGTGATG-3ʹ; Ufd1-F: 5ʹ-TACTCGAGTTTGTGGCCGAC-3ʹ; Ufd1-R: 5ʹ-AGCACCTTCTTAACGACCGG-3ʹ; TPBH-F: AAGTACCGCAACCTGGACAC-3ʹ; TBPH-R: 5ʹ-CGGGATCAAGGAAGGTTACA-3ʹ; Futsch-F: 5ʹ-GCGAGTGCATCAACGGATTC-3ʹ Futsch-R: 5ʹ-GTTAAAGTCGCGCGAATCCC-3ʹ. PCR conditions were 95°C for 30 s, 60°C for 30 s and 72°C for 1 min, repeated 20 cycles for *Rp49*, 29 cycles for *Npl4* and *Ufd1*, 26 cycles for *TBPH* and 35 cycles for *Futsch*. Quantification analysis was conducted on at least five different samples for each genotype. PCR products were run on ethidium bromide containing agarose gel for visualisation and band intensities were quantified using ImageJ (ImageJ; NIH, Bethesda, USA). *Rp49* intensities were used as control for cDNA concentration in the PCR reaction.

### 4.4 Immunoblot test

Soluble protein lysate was purified from L3 stage larvae as described previously (O'Sullivan et al., 2013). Samples were run on 10% acrylamide separating gels and transferred to nitrocellulose membrane (GE Healthcare Life Sciences, Buckinghamshire, UK) by electrophoresis. After blocking in 5% non-fat milk in 10 mM Tris-HCL, 100 mM NaCl and 0.05% Tween-20 (TBS-T) for 1 h, the membrane was then subjected to overnight incubation with primary antibodies against Xbp-1 (M-186; Santa Cruz Biotechnology, Heidelberg, Germany) and α-tubulin (T9026; Sigma, Poole, UK) at 4°C. Following incubation with HRP (horseradish peroxidase)-coupled secondary antibody for 1 h at room temperature and chemiluminescent substrate (WesternBright ECL; Advansta, USA), the bands were visualised by autoradiography.

### 4.5 Histology and immunomicroscopy

Immunostaining of *Drosophila* larvae was carried out as described previously (Fowler and O'Sullivan, 2016). Briefly, third-instar larvae were dissected in chilled Ca^+2^-free HL3 solution (Stewart et al., 1994), fixed in 4% formaldehyde in PBS for 30 min, permeabilised in 0.1% Triton X-100 in PBS and incubated with primary antibodies against Futsch (22C10; Hybridoma) (Fujita et al., 1982) and HRP (P7899; Sigma). Following incubation with the appropriate secondary antibodies, fixed preparations were mounted in Vectashield containing the nuclear stain DAPI (Vector Laboratories; Peterborough, UK) and were viewed using an Olympus IX81 confocal head mounted on an Olympus Fluoview FV-1000 microscope. All images were acquired using a 60×/1.35NA objective and the FV10-ASW ver.04.01 software. We imaged NMJs at muscle 6/7 of segment A3. Z-stacks of whole VNCs were imaged at 5 μm intervals.

### 4.6 Image analysis

Mean branch length per NMJ was determined in ImageJ, with a branch defined as at least two boutons connected to the NMJ arbor. To quantify tubulin staining within terminal synaptic boutons at NMJs, Futsch staining was classified as present, i.e., detectable staining within terminal bouton stained with Dlg, or absent. Staining was categorized on images in a blinded fashion, without knowing which image corresponded to which sample, and then subsequently ‘un-blinded’.

To measure the intensity of Futsch or mito:GFP staining within axons, the mean grey intensity for axonal regions of interest was quantified using Image J. For analysis of mitochondrial area and circularity, images were i) thresholded and converted to binary images, ii) the watershed function was used on these binarized images to separate adjoined mitochondria, and iii) the mitochondrial area calculated in ImageJ. Mitochondrial circularity was quantified using the Shape Descriptors option in the ImageJ/Analyze menu which uses equation 4 π x [Area]/[Perimeter]^2^ to generate a value between 0 and 1, where 1 is a perfect circle and as the value approaches 0 it becomes increasingly elongated.

For TBPH analysis in VNC, confocal sections were acquired throughout the VNC at 5 μm intervals. For analysis of TBPH-positive puncta, images were: i) thresholded and converted to binary images, ii) the watershed function was used on these binarized images to separate adjoined puncta and the number, and iii) the area of TBPH-positive puncta was quantified in ImageJ.

For all analyses, at least four independent experiments were performed, with at least five larvae per genotype in each experiment.

### 4.7 Statistical analysis

All data were exported to Prism (GraphPad Software, Inc.; La Jolla, CA, USA) for statistical analysis. Statistical significance was determined using one-way ANOVA and Dunnett's post-tests, except for locomotion and lifespan assays. Statistical significance for larval crawling assays was determined using one-way ANOVA and Tukey's post-tests. Statistical significance for adult locomotion assays was determined using two-way ANOVA and Bonferroni post-tests. Lifespan assays were analyzed using the log-rank test.

## Figures and Tables

**FIGS1:**
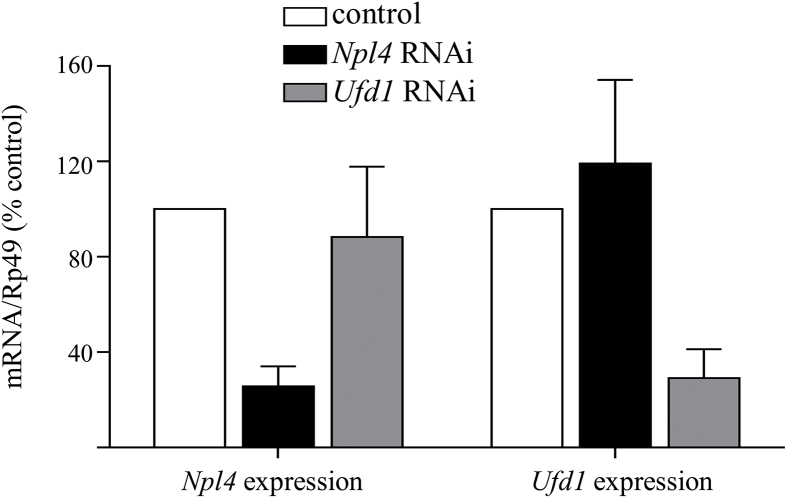


**FIGS2:**
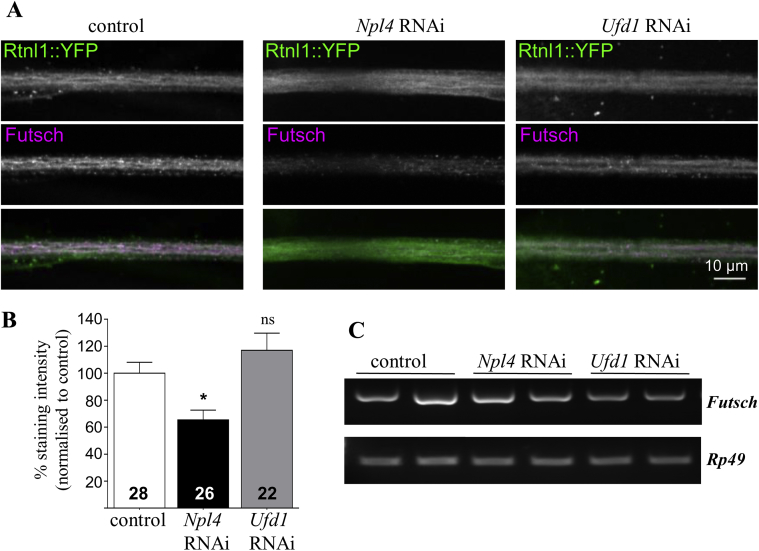


**FIGS3:**
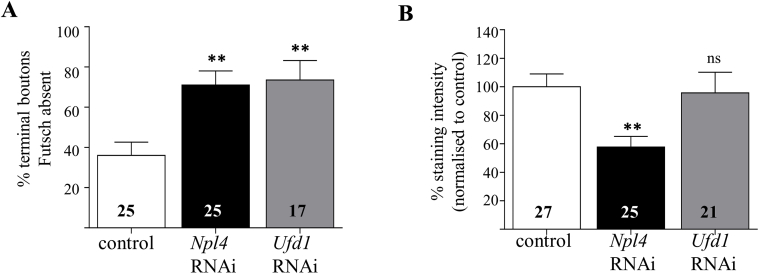


**FIGS4:**