# Supplementary material for: Roles for the VCP co-factors Npl4 and Ufd1 in neuronal function in Drosophila melanogaster
Source: J Genet Genomics. 2017 Oct 20;44(10):493–501. doi: 10.1016/j.jgg.2017.06.003 (PMC5666124; doi:10.1016/j.jgg.2017.06.003)
Supplement: Legend [file mmc1.docx]

**Supplementary** **Figure Legends**

Fig. S1. Analysis of *Npl4* and *Ufd1* expression levels by semi-quantitative PCR. Analysis of *Npl4* and *Ufd1* expression levels in larvae generated by crossing *da-GAL4* to 60100 *w^1118^* (control), *Npl4* RNAi or *Ufd1* RNAi flies. PCR bands were quantified from 5 independent experiments.

Fig. S2. Knockdown of *Npl4* disrupts microtubule expression within axons. **A**: Single confocal sections showing Futsch (magenta) and the ER marker Rtnl1::YFP (green) staining in motor neuron axons. Larvae are progeny of *nSyb-GAL4* crossed to either *w^1118^* (control), *Npl4* RNAi or *Ufd1* RNAi flies. **B**: Graph represents quantification of axonal Futsch staining intensity normalised to control levels with data expressed as mean ± SEM (*n* = 22–26 larvae from 3 independent experiments), and values significantly different from control were determined by one-way ANOVA and Dunnett’s post-tests (ns *P* > 0.05, * *P* < 0.05). **C**: *Futsch* expression globally is not altered by loss of *Npl1* or *Ufd1* as observed by PCR amplification of *Futsch* and *Rp49* cDNA from progeny of *da-GAL4* crossed to either *w^1118^* (control), *Npl4* RNAi or *Ufd1* RNAi flies.

Fig. S3. Disruption of microtubule expression in knockdown of *Npl4* and *Ufd1* posterior motor neurons. **A**: Graph represents percentage of branches in which the terminal bouton of posterior motor neurons are lacking Futsch staining. **B**: Graph represents quantification of axonal Futsch staining intensity within posterior axons normalised to control levels. Data expressed as mean ± SEM (*n* = 17–27 larvae from 3 independent experiments), and values significantly different from control were determined by one-way ANOVA and Dunnett’s post-tests (ns *P* > 0.05, * *P* < 0.05, ** *P* < 0.01).

Fig. S4. Effect of *Npl4* and *Ufd1* knockdown on axonal mitochondria. **A**: Single-confocal sections showing mitochondria (mito::GFP) in the axons of control, *Npl4* RNAi or *Ufd1* RNAi larvae (generated by crossing to the motor-neuron driver *OK6-GAL4*). **B**-**D**: Graphs show quantification of mito::GFP staining intensity (**B**), mitochondrial area (**C**) and mitochondrial circularity (**D**) with data expressed as mean ± SEM with significance determined by one-way ANOVA and Dunnett’s post-tests (*n* = 22–23 larvae from 4 independent experiments; ns *P* > 0.05).
