# Supplementary material for: Roles for the VCP co-factors Npl4 and Ufd1 in neuronal function in Drosophila melanogaster
Source: J Genet Genomics. 2017 Oct 20;44(10):493–501. doi: 10.1016/j.jgg.2017.06.003 (PMC5666124; doi:10.1016/j.jgg.2017.06.003)
Supplement: Tables [file mmc2.docx]

**Supplemental Tables**

**Table S1**. *n* numbers and median lifespan for survival assays.

|  | ***n* number** | **Median life span** |
| --- | --- | --- |
| Control | 89 | 69 |
| *Npl4* RNAi | 168 | 52 |
| *Ufd1* RNAi | 183 | 65 |
| *TBPH* OE | 84 | 36 |
| *Npl4* RNAi *+ TBPH* OE | 83 | 42 |
| *Ufd1* RNAi *+ TBPH* OE | 79 | 40 |
| *TBPH* RNAi | 92 | 46 |
| *Npl4* RNAi *+ TBPH* RNAi | 84 | 32 |
| *Ufd1* RNAi *+ TBPH* RNAi | 92 | 42.5 |

Male flies were generated by crossing *nSyb-GAL4* to *60100 w^1118^* (Control), *Npl4* RNAi, *Ufd1* RNAi, *TBPH* OE*,* *Npl4* RNAi + *TBPH* OE, *Ufd1* RNAi + *TBPH* OE, *TBPH* RNAi*,* *Npl4* RNAi + *TBPH* RNAi and *Ufd1* RNAi + *TBPH* RNAi lines.

**Table S2**. Statistical comparison of survival assays of male flies generated as in Table S1.

| **Log-Rank (Mantel Cox) test** | ***p* value** | ***P* value summary** |
| --- | --- | --- |
| Control *vs Npl4* RNAi | < 0.0001 | *** |
| Control *vs Ufd1* RNAi | 0.3522 | ns |
| Control *vs TBPH* OE | < 0.0001 | *** |
| Control *vs Npl4* RNAi *+ TBPH* OE | < 0.0001 | *** |
| Control *vs Ufd1* RNAi *+ TBPH* OE | < 0.0001 | *** |
| *TBPH* OE *vs Npl4* RNAi *+ TBPH* OE | 0.0125 | * |
| *TBPH* OE *vs Ufd1* RNAi *+ TBPH* OE | 0.0563 | ns |
| *Npl4* RNAi *vs Npl4* RNAi *+ TBPH* OE | < 0.0001 | *** |
| *Ufd1* RNAi *vs Ufd1* RNAi *+ TBPH* OE | < 0.0001 | *** |
| Control *vs TBPH* RNAi | < 0.0001 | *** |
| Control *vs Npl4* RNAi *+ TBPH* RNAi | < 0.0001 | *** |
| Control *vs Ufd1* RNAi *+ TBPH* RNAi | < 0.0001 | *** |
| *TBPH* RNAi *vs Npl4* RNAi *+ TBPH* RNAi | < 0.0001 | *** |
| *TBPH* RNAi *vs Ufd1* RNAi *+ TBPH* RNAi | 0.3910 | ns |
| *Npl4* RNAi *vs Npl4* RNAi *+ TBPH* RNAi | < 0.0001 | *** |
| *Ufd1* RNAi *vs Ufd1* RNAi *+ TBPH* RNAi | < 0.0001 | *** |

**Table S3**. Statistical comparison of larval crawling assays generated as in Table S1.

| **Bonferroni’s multiple comparisons test** | ***P* value summary** |
| --- | --- |
| Control *vs Npl4* RNAi | **** |
| Control *vs Ufd1* RNAi | ** |
| Control *vs TBPH* OE | ns |
| Control *vs Npl4* RNAi *+ TBPH* OE | ns |
| Control *vs Ufd1* RNAi *+ TBPH* OE | **** |
| *TBPH* OE *vs Npl4* RNAi *+ TBPH* OE | ns |
| *TBPH* OE *vs Ufd1* RNAi *+ TBPH* OE | ns |
| *Npl4* RNAi *vs Npl4* RNAi *+ TBPH* OE | ns |
| *Ufd1* RNAi *vs Ufd1* RNAi *+ TBPH* OE | ns |
| Control *vs TBPH* RNAi | ns |
| Control *vs Npl4* RNAi *+ TBPH* RNAi | *** |
| Control *vs Ufd1* RNAi *+ TBPH* RNAi | **** |
| *TBPH* RNAi *vs Npl4* RNAi *+ TBPH* RNAi | ns |
| *TBPH* RNAi *vs Ufd1* RNAi *+ TBPH* RNAi | ns |
| *Npl4* RNAi *vs Npl4* RNAi *+ TBPH* RNAi | ns |
| *Ufd1* RNAi *vs Ufd1* RNAi *+ TBPH* RNAi | ns |

**Table S4**. Statistical comparison of climbing assays of male flies generated as in Table S1.

| **Bonferroni’s multiple comparisons test** | ***P* value summary** |
| --- | --- |
| Control *vs Npl4* RNAi | **** |
| Control *vs Ufd1* RNAi | **** |
| Control *vs TBPH* OE | **** |
| Control *vs Npl4* RNAi *+ TBPH* OE | **** |
| Control *vs Ufd1* RNAi *+ TBPH* OE | **** |
| *TBPH* OE *vs Npl4* RNAi *+ TBPH* OE | ns |
| *TBPH* OE *vs Ufd1* RNAi *+ TBPH* OE | ns |
| *Npl4* RNAi *vs Npl4* RNAi *+ TBPH* OE | ns |
| *Ufd1* RNAi *vs Ufd1* RNAi *+ TBPH* OE | ** |
| Control *vs TBPH* RNAi | *** |
| Control *vs Npl4* RNAi *+ TBPH* RNAi | **** |
| Control *vs Ufd1* RNAi *+ TBPH* RNAi | **** |
| *TBPH* RNAi *vs Npl4* RNAi *+ TBPH* RNAi | **** |
| *TBPH* RNAi *vs Ufd1* RNAi *+ TBPH* RNAi | **** |
| *Npl4* RNAi *vs Npl4* RNAi *+ TBPH* RNAi | **** |
| *Ufd1* RNAi *vs Ufd1* RNAi *+ TBPH* RNAi | **** |
